# A review of perioperative treatment strategies with immunotherapy and tyrosine kinase inhibitors in resectable and stage IIIA-N2 non-small cell lung cancer

**DOI:** 10.3389/fonc.2024.1373388

**Published:** 2024-03-27

**Authors:** Madeleine B. Hopson, Sawsan Rashdan

**Affiliations:** ^1^ Division of Hematology and Oncology, Department of Internal Medicine, University of Texas Southwestern Medical Center, Dallas, TX, United States; ^2^ Harold C. Simmons Comprehensive Cancer Center, University of Texas Southwestern Medical Center, Dallas, TX, United States

**Keywords:** lung cancer, immunotherapy, tyrosine kinase inhibitors, adjuvant, neoadjuvant, early-stage, resectable

## Abstract

Stage IIIA-N2 non-small cell lung cancer (NSCLC) is a heterogeneous group with different potential therapeutic approaches. Treatment is typically multimodal with either surgical resection after neoadjuvant chemotherapy and/or radiation or concurrent chemotherapy and radiation if unresectable. Despite the multimodal treatment and early stage, cure rates have traditionally been low. The introduction of immunotherapy changed the treatment landscape for NSCLC in all stages, and the introduction of immunotherapy in early-stage lung cancer has improved event free survival and overall survival. Tyrosine Kinase inhibitors (TKIs) have also improved outcomes in early-stage mutation-driven NSCLC. Optimal treatment choice and sequence is increasingly becoming based upon personalized factors including clinical characteristics, comorbidities, programmed death-ligand 1 (PD-L1) score, and the presence of targetable mutations. Despite encouraging data from multiple trials, the optimal multimodal sequence of stage IIIA-N2 NSCLC treatment remains unresolved and warrants further investigation. This review article summarizes recent major clinical trials of neoadjuvant and adjuvant treatment including stage IIIA-N2 NSCLC with a focus on immunotherapy and TKIs.

## 1 Introduction

Lung cancer was the third most diagnosed cancer and the cancer with the highest mortality rate in 2020 (1). Stage IIIA-N2 NSCLC is considered regional disease, which comprises 21% of new lung cancer diagnoses, with a 5-year overall survival (OS) rate of 34.8% (2). Optimal management of stage IIIA-N2 non-small cell lung cancer (NSCLC) has recently evolved into a heterogeneous group of treatment strategies. Stage IIIA-N2 NSCLC is classified as a primary lung tumor ≤ 5 cm in its greatest dimension with no further metastases than the ipsilateral mediastinal and/or subcarinal lymph nodes. At presentation, this group of tumors is classified as either incidental N2 discovered during surgery, potentially resectable after induction therapy, or unresectable. Unresectable tumors are generally tumors with bulky N2 involvement or with T4 involvement (tumor size > 7 cm, direct tumor invasion of surrounding anatomical structures, or a separate tumor in a different lobe of the ipsilateral lung) (3). Tumor resectability is typically determined within a multidisciplinary committee. Based on current NCCN guidelines, peri-operative management consists of 3-4 cycles of neoadjuvant platinum-doublet chemotherapy (with immunotherapy in patients who are candidates for immune checkpoint inhibitors), followed by adjuvant chemotherapy, and subsequent immunotherapy or tyrosine kinase inhibitor (TKI) in appropriate candidates (4). With the introduction of immunotherapy and targeted molecular therapies, the choice of which agents to select for these treatment modalities remains debated. In addition to OS, event-free survival (EFS), and disease-free survival (DFS), major pathologic response (MPR) is also used to determine which combination of these modalities is most beneficial. MPR is traditionally defined as the presence of ≤ 10% viable tumor after surgical resection and is frequently used as a surrogate marker for OS (5).

Immunotherapy first revolutionized treatment for NSCLC in the metastatic setting and has progressively been incorporated into all stages of treatment (6). Current immunotherapy agents used in NSCLC inhibit the checkpoint proteins that down-regulate T cells, including programmed death-1 (PD-1)/PD-L1 and cytotoxic T-lymphocyte associated protein 4 (CTLA-4) (7). PD-1 and PD-L1 inhibitors have traditionally been used independently, or in combination with CTLA-4 inhibitors due to improved survival outcomes (8). TKIs target certain driver mutations in protein kinases that promote tumor growth, progression, and regulation (9). Some of the most common and targetable mutations in NSCLC include epidermal growth factor receptor (*EGFR*) mutations, anaplastic lymphoma kinase (*ALK*) rearrangements, *ROS1* rearrangements, rearranged during transfection (*RET*) rearrangements, *BRAF* mutations, and Kirsten rat sarcoma viral oncogene homolog (*KRAS*) mutations (10).

This review article will summarize the relevant clinical trials that include treatment for stage IIIA-N2 resectable or potentially resectable NSCLC. It will examine the different immunotherapy and TKI treatment options with the goal of identifying patients in this stage who may benefit from these regimens.

## 2 Methods

Included articles were found through a review of databases, such as PubMed and Google Scholar. Search terms included, “stage IIIA non-small cell lung cancer,” “stage IIIA-N2 non-small cell lung cancer,” “perioperative immunotherapy in stage IIIA-N2 non-small cell lung cancer,” “adjuvant tyrosine kinase inhibitors in stage IIIA-N2 non-small cell lung cancer,” and “neoadjuvant tyrosine kinase inhibitors in stage IIIA non-small cell lung cancer”. Prior review articles on the topic also served as a substrate for compiling the major clinical trials referencing this topic. All studies that had outcome evaluations of perioperative chemotherapy in combination with or compared to immunotherapy or TKIs in patients with resectable, stage III NSCLC were included. Included studies were not limited to patients only with stage IIIA-N2 NSCLC as long as this subgroup was included in the overall outcome.

## 3 Neoadjuvant strategies

### 3.1 Neoadjuvant immunotherapy

The safety and feasibility of neoadjuvant nivolumab was initially shown in patients with resectable, stage I-IIIA NSCLC with the CheckMate 159 study published in 2018. Nivolumab was given at a dose of 3 mg/kg of body weight every 2 weeks for 2 cycles and achieved a MPR in 9 (45%) of 20 resected tumors (11). MPR was seen almost equally in both PD-L1 positive and negative tumors, although some of the PD-L1 negative tumors had infiltrating immune cells that were PD-L1 positive, suggesting a possible predilection for PD-L1.

Since then, multiple other studies have evaluated the safety and efficacy of neoadjuvant immunotherapy in resectable NSCLC with variable but encouraging results (**Table 1**) (12). The ChiCTR-OIC-17013726 trial (2020) had similar MPR rates with 2 cycles of neoadjuvant sintilimab with 15 (40.5%) of 37 patients with stage IA-IIIB resectable NSCLC achieving a MPR (13). Interestingly, the MPR was only seen in patients with squamous cell NSCLC compared to adenocarcinoma (MPR 48.4% vs. 0%, respectively). However, sintilimab is not currently approved for use in the United States (14). In the LCMC3 trial (2021), 2 cycles of neoadjuvant atezolizumab achieved a MPR in 30 (20%) of 147 patients with stage IB-IIIB resectable NSCLC (15). Despite the lower MPR rate in the atezolizumab study, patients experienced no major delays to surgery, only 5% of patients had grade ≥3 treatment-related adverse events (TRAEs), and the 3-year OS rate was 80% (16). In the IONESCO (IFCT-1601) trial (2022), 3 cycles of neoadjuvant durvalumab (750 mg every 2 weeks) resulted in a MPR in 8 (18.6%) of 43 patients with stage IB-IIIA, resectable NSCLC, with a 12-month median OS rate of 78% (17). In the TOP1501 phase II trial (2022), patients with stage IB-III resectable NSCLC who received 2 cycles of pembrolizumab (200 mg) had a MPR in 7 (28%) of 25 patients, primarily occurring in patients with adenocarcinoma histology (18). The NEOMUN trial is a recently completed trial with results still pending that also evaluated neoadjuvant pembrolizumab, but specifically in patients with stage II/IIIA resectable NSCLC (19).

**Table 1:** Review of neoadjuvant immunotherapy trials.

Trial	Phase	NSCLC Stage	Treatment Arm	Number of Cycles	Comparison Arm	MPR (%)	PCR (%)	Grade≥3 AEs (%)
IONESCO (2022) *Wislez et al.*	2	IB-IIIA	Durvalumab	3	–	18.6	17	11
LCMC3 (2021) *Chaft et al.*	2	IB-IIIB	Atezolizumab	2	–	20	6	5
TOP1501 (2022) *Tong et al.*	2	IB-III	Pembrolizumab	2	–	28	12	–
NEOSTAR (2021) *Cascone et al.*	2	I-IIIA	Nivolumab and Ipilimumab	3	Nivolumab	38	29	10
ChiCTR-OIC-17013726 (2020) *Gao et al.*	1b	IA-IIIB	Sintilimab	2	–	40.5	16.2	10
CheckMate 159 (2018) *Forde et al.*	Pilot	I-IIIA	Nivolumab	2	–	45	10	4.5

NSCLC, Non-small cell lung cancer; MPR, Major pathologic response; PCR, pathologic complete response; AE, adverse event.

–, no data.

In 2021, the NEOSTAR trial was the first trial to test combination neoadjuvant immunotherapy. It showed that neoadjuvant nivolumab and ipilimumab in patients with stage I-IIIA resectable NSCLC had a greater number of patients with a MPR than with neoadjuvant nivolumab alone [8 (38%) of 21 patients vs. 5 (22%) of 23 patients, respectively] (20). Six (29%) of 21 patients in the combination group achieved a pathologic complete response (PCR). Disease stage was not significantly related to recurrence-free survival (RFS) rates, but patients with stage IIIA disease had worse lung-cancer related RFS rates compared to stage I or II disease. Nivolumab was given at a dose of 3 mg/kg every 2 weeks for 3 doses and ipilimumab was given at a dose of 1 mg/kg for 1 dose. Importantly, grade 3-5 immune-related adverse events (IRAEs) only occurred in 2 (10%) of 21 patients given both nivolumab and ipilimumab, which was similar to the IRAE rate of nivolumab alone. PD-L1 expression correlated with improved pathologic responses, but there were many tumors without PD-L1 expression that also showed a pathologic response. This suggests PD-L1 expression is a sufficient but not necessary characteristic for treatment response.

### 3.2 Neoadjuvant immunotherapy and chemotherapy

Historically, induction chemotherapy alone has resulted in a median survival rate of 15.9 to 33 months in stage III NSCLC, with the addition of induction radiotherapy not significantly benefiting median EFS (21).

In 2020, the NADIM trial was one of the first trials to show that the addition of 3 cycles of neoadjuvant nivolumab with platinum-based chemotherapy followed by adjuvant nivolumab for 1 year had a progression-free survival of 77.1% at 2 years in patients with resectable stage IIIA NSCLC (22). OS data at 3 years follow-up is encouraging at 81.9% (23). 34 (83%) of 41 patients achieved a MPR, with 26 (63%) patients achieving a PCR. Patients with a PD-L1 tumor proportion score (TPS) ≥ 25% were more likely to have a MPR or PCR, but 58% of patients with a PD-L1 TPS <25% still had a MPR or PCR, suggesting PD-L1 was not a sensitive marker of response. PD-L1 expression was also not associated with OS. Additionally, *STK11* and *EGFR* mutations were associated with reduced PFS.

Similar results were seen in the Columbia trial, a phase II trial in which patients with stage IB-IIIA NSCLC were given up to 4 cycles of neoadjuvant atezolizumab and platinum-based chemotherapy. While not exclusively focusing on stage IIIA disease, 23 (77%) of the 30 patients included had stage IIIA disease. In this study, 17 (57%) of 30 patients achieved a MPR, with 10 (33%) of 30 patients achieving a PCR (24). Of the 10 patients who achieved a PCR, 6 of them had stage IIIA disease at presentation. No significant association was seen between PD-L1 expression and MPR or PCR. However, a *post hoc* analysis showed a median best percentage change of MPR of 6% in patients with a PD-L1 expression of ≥1% (40% vs. 34% in patients with PD-L1 expression < 1%), however this finding was non-significant. *Post hoc* analyses also showed more patients with squamous histology than adenocarcinoma histology had a MPR and PCR, none of the patients with *STK11* tumor mutations had any response, and 2 out of 4 patients with *EGFR* mutations had PCRs.

The SAKK 16/14 trial (2021) showed that neoadjuvant durvalumab and chemotherapy in patients with stage IIIA-N2 NSCLC had a MPR in 34 (62%) of 55 patients, a PCR in 10 (18%) of 55 patients, and a 1-year EFS of 73% (25). There were no significant effects of PD-L1 expression on MPR, nodal downstaging, or 1-year EFS. Fifty-five (82%) of 67 patients made it to surgery, with 3 (4%) patients unable to make it to surgery because of treatment discontinuation due to toxicity.

The CheckMate 816 trial (2022) was one of the first trials to compare the use of neoadjuvant immunotherapy and chemotherapy to the traditional use of neoadjuvant chemotherapy alone. In this randomized, phase III trial, patients with stage IB-IIIA resectable NSCLC who received neoadjuvant nivolumab and platinum-based chemotherapy had a greater MPR (36.9% vs. 8.9%), greater PCR (24% vs. 2.2%), longer median EFS (31.6 vs. 20.8 months), and higher 2-year EFS (63.8% vs. 45.3%) compared to neoadjuvant chemotherapy alone (26). This benefit was greatest in patients with stage IIIA disease (HR for disease progression, recurrence, or death of 0.54), a tumor PD-L1 expression of ≥ 1% (MPR of 44.9%, PCR of 32.6%), and in patients with non-squamous histology. 83.2% of the patients in nivolumab-plus-chemotherapy group underwent surgery, which was not statistically different than in the chemotherapy-alone group (75.4%). Patients in the combination group had fewer overall AEs (92.6% vs. 97.2%), fewer grade ≥ 3 TRAEs (33.5% vs. 36.9%), fewer AEs leading to surgical delays (3.4% vs. 5.1%), shorter median durations of surgery, more commonly used minimally invasive surgical approaches, and fewer pneumonectomies compared to the chemotherapy alone group. In a recently published 3-year follow-up exploratory analysis, patients who were treated with neoadjuvant nivolumab and chemotherapy had 3-year OS rates of 85% (vs. 66% with chemotherapy alone; however, this data was a trend as the OS was still immature) and 3-year EFS rates of 57% (vs. 43% with chemotherapy alone) (27). Higher PD-L1 expression continued to demonstrate increased benefits with patients with PD-L1 expression ≥ 1% achieving a 3-year OS of 85% and a 3-year EFS of 72% (compared to 66% and 47%, respectively, with chemotherapy alone) (28). This trial led to the FDA approval of neoadjuvant nivolumab with platinum-doublet chemotherapy in early-stage NSCLC (29).

The AEGEAN trial (2023) was a randomized, phase III trial that showed that patients with stage II-IIIB (N2) resectable NSCLC who received 4 cycles of neoadjuvant durvalumab with chemotherapy followed by 1 year of adjuvant durvalumab had a higher PCR (17.2% vs. 4.3%) and increased 2-year EFS (63.3% vs. 52.4%) compared to neoadjuvant chemotherapy alone (30). At baseline, 338 (45.7%) of 740 patients in the trial had stage IIIA disease. Patients with all levels of PD-L1 expression derived benefit from the durvalumab and chemotherapy combination, but the magnitude of benefit was greatest in patients with PD-L1 expression ≥ 50%. The benefit was also greatest in current and former smokers. Patients with stage IIIA disease who received durvalumab and chemotherapy had the greatest EFS benefit. The trial had 51 patients with known *EGFR* mutations but a subgroup analysis showed no clear evidence of clinical benefit with durvalumab compared to placebo (however, the subgroup analysis was not sufficiently powered) (31).

The NADIM II trial (2023) was a randomized phase II trial that showed that patients with resectable stage IIIA or IIIB NSCLC who received neoadjuvant nivolumab and platinum-based chemotherapy compared to neoadjuvant chemotherapy alone had a greater PCR (37% vs. 7%), greater MPR (53% vs. 14%), overall response (75% vs. 48%), improved 2-year PFS (67.2% vs. 40.9%), and greater 2-year OS (85% vs. 63.6%) (32). Patients who were treated with the combination neoadjuvant nivolumab and chemotherapy and who had PD-L1 expression of ≥ 1% had the greatest improvement in PCR rates. Neoadjuvant nivolumab and chemotherapy also resulted in more patients undergoing surgery than in the chemotherapy-alone group (93% vs. 69%). Notably, the NADIM II trial used a carboplatin dose of area under the concentration-time curve of 5 mg/ml, compared to the 6 mg/ml used in the NADIM trial.

The KEYNOTE-671 trial (2023) was a randomized phase III trial that showed that patients with resectable stage II-IIIB (N2) NSCLC who received 4 cycles of neoadjuvant pembrolizumab (200 mg every 3 weeks) and cisplatin-based chemotherapy, followed by surgery and up to 13 cycles adjuvant pembrolizumab had an improved 2-year EFS (62.4% vs. 40.6%), higher MPR (30.2% vs. 11%), and higher PCR (18.1% vs. 4%) compared to patients who received only neoadjuvant cisplatin with placebo, followed by surgery and adjuvant placebo (33). The EFS benefit was generally consistent across most subgroups, except for lack of significance in the PD-L1 expression < 1%, pathologic stage II, and never smoker subgroups; however, many of these subgroup analyses were not adequately powered. This suggests that patients with stage III disease derived more benefit from neoadjuvant chemoimmunotherapy than patients with stage II disease. This study also did not find a difference in the benefit of pembrolizumab between squamous and non-squamous histology. Increased EFS was also seen in patients in the pembrolizumab group who did not have a MPR or PCR, suggesting that adjuvant pembrolizumab provided further benefit. OS was not significant in the interim analysis. The addition of pembrolizumab to neoadjuvant chemotherapy did not affect the ability to undergo surgery or increase the rate of surgical complications.

An initial update from the CheckMate 77T trial (2023), a randomized, phase III trial, showed that patients with stage IIA-IIIB resectable NSCLC who received 4 cycles of neoadjuvant nivolumab and platinum-doublet chemo followed by surgery and adjuvant nivolumab for 1 year had improved median EFS (not reached vs. 18.4 months), 18-month EFS (70% vs. 50%), PCR (25.3% vs. 4.7%), and MPR (35.4% vs. 12.1%) compared to patients who received 4 cycles of neoadjuvant platinum-doublet chemotherapy with placebo, followed by surgery and adjuvant placebo for 1 year (34). Benefit was most pronounced in patients with stage III disease, PD-L1 ≥ 1%, current and former smokers, those with squamous histology (35). Definitive surgery rates were similar between both groups (36).

A summary of the reported trials is shown in **Table 2**. There are additional ongoing trials looking at the addition of immunotherapy to neoadjuvant chemotherapy. The IMpower030 trial is an ongoing randomized, phase III trial looking at the effects of neoadjuvant atezolizumab plus platinum-based chemotherapy followed by up to 16 cycles of adjuvant atezolizumab compared to neoadjuvant platinum-based chemotherapy plus placebo followed best supportive care in patients with stage II-IIIB resectable NSCLC (37). This trial is estimated to be completed in 2025.

**Table 2:** Review of neoadjuvant immunotherapy and chemotherapy trials.

Trial	Phase	NSCLC Stage	Treatment Arm	Number of Cycles	Comparison Arm	MPR (%)	PCR (%)	EFS (%)	OS (%)	PD-L1 Conclusions	EGFR Benefit	Grade ≥3 AEs (%)
AEGEAN (2023) *Heymach et al.*	3	II-IIIB	Durvalumab + platinum-based chemotherapy	NA: 4 A: 12	Placebo + platinum-based chemotherapy	33.3	17.2	1-yr:73.4 2-yr:63.3	–	Most Benefit: PD-L12 ≥ 50%	N	42.4
KEYNOTE 671 (2023) *Wakelee et al. *	3	II-IIB	Pembrolizumab + Cisplatin-based chemotherapy	NA: 4 A: 13	Placebo + Cisplatin-based chemotherapy	30.2	18.1	2-yr:62.4	–	Most Benefit: PD-L1 ≥ 50% No Benefit: PD-L1 < 1%	–	44.9
CheckMate 77T (2023) *Cascone et al. *	3	IIA-IIIB	Nivolumab + platinum-doublet chemotherapy	NA: 4 A: 1 yr	Placebo + platinum-doublet chemotherapy	35.4	25.3	1.5-yr:70	–	Most Benefit: PD-L1 ≥ 1%	–	32
CheckMate 816 (2022) *Forde et al.*	3	IB-IIIA	Nivolumab + platinum-doublet chemotherapy	NA: 3	Platinum-doublet chemotherapy	36.9	24	1-yr:76.1 2-yr:63.8 3-yr:57	3-yr: 85	Most Benefit: PD-L1 ≥ 21%	–	33.5
NADIM II (2023) *Provencio et al.*	2	IIIA-IIIB	Nivolumab + Carboplatin (AUC5) Paclitaxel (200 mg/m2)	NA: 3 A: 6 mo	Carboplatin (AUC5 Paclitaxel (200 mg/m2)	53	37	2-yr:67.2	2-yr:85	Most Benefit: PD-L1 ≥ 1%	–	19
COLUMBIA (2020) *Shu et al.*	2	IB-IIIA	Atezolizumab + Carboplatin (AUC5) Nab-paclitaxel (100 mg/m2)	NA: 4	–	57	33	–	–	NS	–	50
SAKK 16/14 (2021) *Rothschild et al.*	2	IIIA(N2)	Durvalumab + Cisplatin (100 mg/m2) + Docetaxel (85 mg/m2)	NA: 3 A: 1 yr	–	62	18	1-yr:73 2-yr:68	1-yr:91 2-yr:83	NS	–	88
NADIM (2020) *Provencio et al.*	2	IIIA	Nivolumab + Carboplatin (AUC6) Paclitaxel (200 mg/m2)	NA:3 A: 1 yr	–	83	63	2-yr:77.1	1-yr:97.8 2-yr:89.9 1-yr:81.9	Most Benefit: PD-L1 ≥ 25%	N	30

NSCLC, non-small cell lung cancer; MPR, major pathologic response; PCR, pathologic complete response; EFS, event-free survival; OS, overall survival; AE, adverse event; NA, neoadjuvant; A, adjuvant; yr, year; mo, month; N, no; AUC, area under the ROC curve; NS, not significant.

–, no data.

### 3.3 Neoadjuvant immunotherapy and radiation

NCT02904954 (2021) was a randomized, phase II trial that showed that patients with stage I-IIIA resectable NSCLC who were treated with 2 cycles of neoadjuvant durvalumab and stereotactic body radiotherapy (8 Gy x 3 fractions) had a 16-fold increased odds of achieving MPR than with neoadjuvant durvalumab alone (ORR 16.0; p<0.0001) (38). Sixteen (53.3%) of 30 patients who received the neoadjuvant radiotherapy and durvalumab achieved an MPR vs. 2 (6.7%) of 30 patients in the durvalumab-alone group. Pathologic CR was seen in 8 (26.7%) of the 30 patients in the combination group. AEs were slightly higher in the combination group with 6 (20%) of 30 patients having a grade ≥ 3 AE compared to 5 (17%) of 30 patients in the durvalumab-only group. No treatment-related deaths were reported.

However, CASE 4516 was a phase I trial looking at the safety and feasibility study of neoadjuvant chemotherapy, radiation, and pembrolizumab in patients with stage IIIA, resectable NSCLC, but the study was terminated early due to a higher-than-expected toxicity rate (39).

There are other ongoing trials evaluating the combination of neoadjuvant radiation, chemotherapy, and/or immunotherapy. NCT03237377 is a phase II trial looking at the toxicity, feasibility, and response rate of 2 cycles of neoadjuvant durvalumab with concurrent standard thoracic radiation (45Gy in 25 fractions) in patients with resectable NSCLC (40). The study is expected to be completed in 2024. The SAKK 16/18 trial is a phase II trial comparing response and survival outcomes in patients with Stage III-N2 resectable NSCLC who receive neoadjuvant chemotherapy, neoadjuvant immunotherapy, or neoadjuvant immuno-radiotherapy (41). Patients assigned to the immuno-radiotherapy arm will be randomly assigned to 3 different fractionation regimens to assess optimal dosing. This study is expected to be completed in 2025.

### 3.4 Neoadjuvant TKIs

A summary of the reported neoadjuvant TKI trials is seen in **Table 3**. In 2019, the ML25444 trial was one of the first phase II trials to test neoadjuvant *EGFR* TKIs in stage IIIA-N2 *EGFR*-mutated NSCLC. Nineteen patients received erlotinib 150 mg daily for 8 weeks prior to surgery and 13 patients (68.4%) achieved an R0 resection (42). Five (35.7%) of 14 patients with resectable tumors had pathologic downstaging from N2 to N1 or N0. AEs occurred in 7 (36.8%) of patients, with only one patient experiencing a grade 4 AE (hepatitis), and no treatment-related deaths. This study suggested that neoadjuvant TKIs could increase the number of patients with R0 resections and nodal downstaging.

**Table 3:** Review of neoadjuvant TKI trials.

Trial	Phase	NSCLC Stage	Treatment Arm (mutation target)	Treatment Time	Comparison Arm	Adjuvant Treatment	Pathologic Downstaging (%)	R0 Resection Rate (%)	MPR (%)	PCR (%)	ORR (%)	Median PFS (months)	AEs (%)
ML25444 (2019) *Xionget al.*	2	IIIA(N2)	Erlotinib (EGFR)	8 weeks	–	Platinum- based chemotherapy	35.7	68.4	–	–	42.1	11.2	36.8
EMERGING- CTONG 1103 (2019) *Zhong et al.*	2	IIIA(N2)	Erlotinib (EGFR)	7 weeks	Cisplatin (75 mg/m2) + Gemcitabine (1,250 mg/m2)	Erlotinib (1 year)	10.8	73	9.7	0	54.1	21.5	75.7
NEOS (2023) *Lv et al.*	2b	IIA-IIIB	Osimertinib (EGFR)	6 weeks	–	–	46.9	93.8	10.7	3.6	71.1	–	75
NCT03433469 (2023) *Aredoet al.*	2	I-IIIA	Osimertinib (EGFR)	8 weeks	–	–	44	89	15	0	46	32	11
ASCENT (2021) *Piper-Vallillo et al.*	2	IIIA/IIIB	Afatinib (EGFR) + Cisplatin (75 mg/m2) + Pemetrexed (500 mg/m2) + RT +/- surgery	8 weeks	–	Afatinib (2 years)	11.1	–	70	10	58	34.6	47

TKI, tyrosine kinase inhibitor; NSCLC, Non-small cell lung cancer; MPR, Major pathologic response; PCR, pathologic complete response; ORR, objective response rate; PFS, progression-free survival; AE, adverse event.

–, no data.

The EMERGING-CTONG 1103 trial (2019) was a randomized, phase II trial evaluating 6 weeks of neoadjuvant erlotinib vs. 2 cycles of chemotherapy (gemcitabine 1,250 mg/m^2^ and cisplatin 75 mg/m^2^) in patients with stage IIIA-N2 NSCLC with *EGFR* exon 19 or 21 mutations, followed by 12 months of adjuvant erlotinib vs. 2 cycles of adjuvant chemotherapy (gemcitabine 1,250 mg/m^2^ and cisplatin 75 mg/m^2^). Neither neoadjuvant erlotinib nor chemotherapy showed a significant ORR and neither treatment achieved a PCR. MPR was only seen in 3 (9.7%) of the 21 patients in the erlotinib arm (compared to 0% of patients in the chemotherapy arm) (43). The erlotinib group did show a significantly longer median PFS (21.5 months vs. 11.4 months) compared to the chemotherapy group. Grade ≥ 3 AEs occurred in 5 (13.5%) of 37 patients in the erlotinib group vs. 10 (29.4%) of 34 patients in the chemotherapy group. The lack of significant OS benefit in this trial may be related to the inclusion of potentially resectable patients as opposed to clearly resectable patients that are typically used in other neoadjuvant trials. Studies in metastatic NSCLC have also shown that the generation of TKI affects survival outcomes, with osimertinib providing more benefit than erlotinib (44). However, the EMERGING-CTONG 1103 study does suggest that in patients with *EGFR* mutations, there is no apparent increased OS benefit of neoadjuvant chemotherapy, and that neoadjuvant TKIs show an improved PFS benefit compared to neoadjuvant chemotherapy, although this finding was not significant. These findings, along with a greater number of grade ≥ 3 AEs associated with neoadjuvant chemotherapy, suggest that treatment with a neoadjuvant TKI rather than chemotherapy might lead to the same, if not better, outcomes with decreased treatment-related toxicity.

The ASCENT trial (2021) was a phase II study that showed that potentially resectable stage IIIA patients who received 2 months of neoadjuvant afatinib and radiotherapy (median dose 54 Gy) had an ORR of 58% (11 of 19 patients), a MPR in 7 (70%) of 10 patients, a PCR in 1 (10%) of 10 patients, a median PFS of 34.6 months, and 2-year OS of 88% (45). One of the patients who was initially deemed inoperable became operable after response to neoadjuvant afatinib. Patients also continued either adjuvant afatinib or definitive chemoradiation. The most common grade ≥3 toxicities included rash and diarrhea and 5 patients (26%) needed to dose-reduce afatinib (46).

The NEOS trial (2023) was a phase II trial evaluating neoadjuvant osimertinib in *EGFR*-mutated, resectable, stage IIA-IIIB NSCLC. At baseline, 22 (55%) of the 40 patients in the trial had stage IIIA disease. Mutations were limited to *EGFR* exon 19 and/or 21 mutations. Forty patients were assigned to receive 6 weeks of neoadjuvant osimertinib (80 mg daily) with an overall response rate of 71.1%, an R0 resection in 93.8% of patients, an MPR in 10.7% of patients, and a PCR in 3.6% of patients (47). Seven (41.2%) of 17 resected patients with baseline N2 disease were downstaged to N1 or N0 after neoadjuvant Osimertinib. AEs occurred in 82.5% of patients, most commonly rash, diarrhea, and oral ulcerations. Three (7.5%) of the 40 patients had grade 3 AEs, including rash, hypertension, and nephrotic syndrome. No grade 4 or 5 AEs occurred, and no patients had to dose-reduce osimertinib. Sixteen percent of patients did not make it to surgery, primarily due to patient preference.

NCT03433469 (2023) was a phase II trial that showed that patients with resectable, stage I-IIIA *EGFR*-mutated NSCLC who received up to 8 weeks of neoadjuvant osimertinib (80 mg daily) had a MPR in 4 (15%) of 27 patients, 0 PCRs, and nodal downstaging in 4 (44%) of 9 patients (48). Median DFS was 32 months with immature OS data. There were two grade 3 AEs (pulmonary embolism and atrial fibrillation) (49). The study also noted that in 4 of 6 patients who did not achieve a pathologic response, there was a loss of function mutation in *RBM10* (50).

The NeoADAURA trial is an ongoing, phase III trial that will look at MPR and survival outcomes in patients with stage II-IIIB, N2, *EGFR*-mutated NSCLC who received neoadjuvant osimertinib with or without chemotherapy (51). Patients in the osimertinib arm will receive 9 weeks of osimertinib compared to the 6 weeks received in the NEOS trial (52). Estimated completion date is in 2029. The NAUTIKA1 trial is an ongoing, phase II trial that will look at the efficacy of targeted therapies in the neoadjuvant setting for patients with stage IB-IIIB NSCLC with *ALK*, *ROS1*, *NTRK*, *BRAF*, *RET*, *KRAS G12C* mutations (53). It is expected to be completed in 2029.

## 4 Adjuvant strategies

### 4.1 Adjuvant immunotherapy

The IMpower010 study (2021) was a randomized, phase III study assessing the effects of 1 year of adjuvant atezolizumab after 1 to 4 cycles of adjuvant cisplatin-based chemotherapy in patients with stage IB-IIIA, completely resected NSCLC. At baseline, 413 (47%) of 882 patients studied in the trial had stage IIIA disease. In all patients with stage II-IIIA NSCLC, especially those with a PD-L1 expression ≥ 1%, adjuvant atezolizumab reduced the risk of recurrence or death by 34% (21% if not stratified by PD-L1 expression) compared to observation (54). Follow-up data published in 2023 shows that at a median duration of follow-up of 45.3 months, 25% of the patient population had died. In patients who had died, disease progression was the primary cause of death in 63% of the patients in the atezolizumab group and 80% of the patients in the observation group; the median OS did not meet significance and the OS remains immature (55). *Post hoc* exploratory OS analyses showed that patients with PD-L1 expression ≥ 50% had a decreased risk of death by 57% [HR 0.43 (95% CI 0.24-0.78)]. There was no difference in this outcome whether an *EGFR* or *ALK* alteration was present, however this subgroup contained a small number of patients. This study led to the U.S. FDA approval of adjuvant atezolizumab following resection and platinum-based chemotherapy in stage II-IIIA NSCLC with a PD-L1 expression ≥ 1% (56).

The PEARLS/Keynote091 trial (2022) was a randomized, phase III trial, that showed that patients with stage IB-IIIA resected NSCLC and any PD-L1 expression treated with up to 1 year (18 doses) of adjuvant pembrolizumab (200 mg every 3 weeks) after up to 4 cycles of adjuvant chemotherapy had improved median DFS compared to placebo (58.7 vs. 34.9 months) with an estimated 18-month DFS rate of 73.8% (vs. 63.1% in the placebo group) (57). Patients had not received neoadjuvant treatment. Of the 1177 patients enrolled, 339 (29%) had stage IIIA disease at baseline. In patients with PD-L1 expression ≥ 50%, 54 (32%) of 168 patients in the pembrolizumab group had a DFS event (compared to 38% in the placebo group), but median DFS was not reached in either arm (58). Additionally, patients with EGFR mutations who received adjuvant pembrolizumab appeared to have a greater DFS benefit [HR 0.44 (95% CI 0.23-0.84), but the CI overlapped the overall treatment CI, so no definitive conclusion could be drawn. This trial led to the U.S. FDA approval of adjuvant pembrolizumab following resection and platinum-based chemotherapy for stage IB-IIIA NSCLC (59).

There are multiple ongoing trials looking at adjuvant immunotherapy combinations in early-stage, resectable NSCLC. MERMAID-1 is a randomized, phase III trial primarily assessing survival outcomes of patients with stage II-III, resected NSCLC who receive adjuvant chemotherapy with durvalumab or placebo (60). This study was completed in 2023, but results are pending at the time of this review. The ACCIO trial is a randomized, phase III trial comparing survival outcomes in patients with stage IIA-IIIB NSCLC who receive 4 cycles of adjuvant chemotherapy followed by pembrolizumab for 16 to 17 cycles compared to concurrent adjuvant chemotherapy and pembrolizumab for 4 cycles followed by 12 to 13 cycles of pembrolizumab (61). It is estimated to be completed in 2024. The ANVIL trial is a randomized, phase III trial evaluating survival outcomes in patients with stage IB-IIIA, resected NSCLC who receive adjuvant nivolumab for up to 1 year compared to active surveillance (62). This trial is estimated to be completed in 2025. The BR31 trial is a randomized, phase III trial evaluating survival outcomes in patients with IB-IIIA, resected NSCLC who receive adjuvant durvalumab for up to 1 year compared to placebo (63). It is estimated to be completed in 2026.

### 4.2 Adjuvant TKIs

The ADAURA trial (2023) was a randomized, phase III trial, that showed that patients with stage IB to IIIA, resected, *EGFR*-mutated NSCLC who received adjuvant osimertinib 80 mg daily for up to 3 years had a longer DFS at 4 years compared to placebo (73% vs. 38%) and improved 5-year OS compared to placebo (88% vs 78%) (64, 65). Median DFS in the osimertinib group was 65.8 months compared to 28.1 months in the placebo group. This benefit was consistent in all stages (IB through IIIA) and did not differ based on whether the patient received adjuvant chemotherapy (66). The hazard ratio for OS for patients with stage IIIA disease was 0.37 compared to 0.44 and 0.63 in stage IB and II disease, respectively. Regarding CNS benefit, the CNS DFS HR was 0.36 with osimertinib (95% CI, 0.23 to 0.57), with CNS recurrences occurring in 25 (7%) of 339 patients in the osimertinib group and 50 (15%) of 343 patients in the placebo group. AEs occurred in 98% of patients in the osimertinib group vs. 90% of patients in the placebo groups. Diarrhea, paronychia, and dry skin were the most common AEs. Grade ≥ 3 AEs occurred in 23% of the osimertinib group (compared to 14% of the placebo group), with no deaths attributed to osimertinib. Sixty-six percent of patients were able to continue the osimertinib for 3 years. This study led to the U.S. FDA approval of adjuvant osimertinib after surgical resection in patients with NSCLC who have an *EGFR* exon 19 deletion or exon 21 L858R mutation (67).

The TARGET trial is an ongoing phase II trial evaluating the efficacy and safety of adjuvant osimertinib taken for 5 years after surgical resection in patients with stage II-IIIB NSCLC with sensitizing-*EGFR* mutations (68). It will also look at the safety and efficacy of different doses of osimertinib (80 mg vs. 40 mg). It is estimated to be completed in 2029.

There are fewer trials evaluating the optimal treatment for patients with ALK alterations. The ALINA trial is an ongoing, phase III trial evaluating 2 years of adjuvant alectinib vs chemotherapy in *ALK*-rearranged, stage IB-IIIA, resected NSCLC (69). An interim analysis from October 2023 showed that adjuvant alectinib was associated with a significantly higher 3-year DFS than chemotherapy alone (88.7% vs. 54%) and this benefit was consistent in the stage II-IIIA population (70). It is estimated to be completed in 2026. The ALCHEMIST trial also has an ALK-rearranged arm, in addition to an EGFR-mutated arm, primarily looking at improved DFS with TKIs (crizotinib and erlotinib, respectively) vs. placebo in patients with stage IB-IIIA, resected NSCLC that have also completed adjuvant standard of care treatment (71). It is estimated to be completed in 2036 (72).

## 5 Discussion

Treatment of stage IIIA-N2 NSCLC should be individualized and largely include localized treatment with surgery or radiation to cure local disease and systemic therapy to reduce the risk of metastasis. Baseline surgical resectability is also personalized and mainly depends on the patient’s tumor characteristics, nodal involvement, the presence of bulky, infiltrative, or multi-station disease, as well as patient comorbidities and the surgeon’s expertise. The neoadjuvant and adjuvant treatment options for patients with stage IIIA-N2 NSCLC have changed over the last several years with the introduction of immunotherapy. Multiple phase II and III studies have shown benefit of immunotherapy alone or in combination with chemotherapy in early-stage NSCLC, leading to FDA approvals in both the neoadjuvant and adjuvant settings (73). Treatment advancements have improved PCR, MPR, DFS, and EFS, which are increasingly being used as surrogates for OS due to their strong association with improved survival (74, 75). This review article focused on summarizing some of the prominent research trials in this topic over the past few years. It is important to note that none of these studies included baseline unresectable disease with the goal of transforming it to resectable disease.

In the reviewed studies that evaluated neoadjuvant immunotherapy alone, the primary endpoint was MPR, which ranged from 19% with durvalumab to 45% with nivolumab. A meta-analysis looking at MPR rates in patients who underwent neoadjuvant immunotherapy showed a similar MPR of 52% (76). Prior to using immunotherapy in the neoadjuvant setting, the recommendation was to use neoadjuvant chemotherapy for stage IIIA-N2 prior to surgical resection (6). Studies evaluating MPR in neoadjuvant chemotherapy have shown a 22% MPR rate (74). This suggests that neoadjuvant immunotherapy appears to have improved MPR rates compared to traditional neoadjuvant chemotherapy.

The major trials evaluating neoadjuvant chemoimmunotherapy had the highest range of MPR rates, with values between 30.2% in the KEYNOTE 671 trial to 83% in the NADIM trial, which also specifically focused on stage IIIA NSCLC. The NADIM trial also used a carboplatin AUC of 6 mg/ml, which was higher than in the NADIM II trial, which may have improved outcomes with a minimal difference in grade ≥ 3 AEs (30% in the NADIM trial vs. 19% in the NADIM II trial). Also notable is that some of the lower MPR rates were in studies that included stage IIIB patients, including KEYNOTE 671 (MPR of 30.2%) and the CheckMate 77T trial (MPR of 35.4%). The neoadjuvant chemoimmunotherapy trials also focused on EFS and OS. In one prior study evaluating neoadjuvant chemotherapy alone in stage IIIA NSCLC, data showed an improved median OS of 64 months (compared to 11 months with surgery alone) with 2-year and 3-year OS of 60% and 56% respectively (77). In trials focusing on stage III disease, the 2-year EFS ranged from 67.2% in the NADIM II trial to 77.1% in the NADIM trial. The CheckMate 816 trial also showed a 2-year HR for EFS of 0.54 in stage IIIA disease, which was improved over the HR of stage IB or II disease. Regarding OS in trials focusing on stage III disease, the 2-year OS ranged from 83% in the SAKK 16/14 trial to 89.9% in the NADIM trial, with 3-year OS rates of 81.9% also seen in the NADIM trial.

Some of the proposed benefits and risks of using immunotherapy in the neoadjuvant setting are shown in **Table 4** (78–80). In the studies reviewed in this paper, surgeries were not statistically more difficult, did not significantly result in increased post-operative morbidity, and surgical outcomes were overall similar between the neoadjuvant chemoimmunotherapy and chemotherapy alone groups. In the CheckMate 816 trial, pneumonectomies were even performed less frequently in patients given neoadjuvant chemoimmunotherapy than in patients given chemotherapy alone. This finding is consistent with a meta-analysis by Cao et al. looking at neoadjuvant immunotherapy in resectable NSCLC, which showed a lower pneumonectomy rate of 8.6% compared to the 15.8-17.6% rate reported in patients who received neoadjuvant chemotherapy (76). These results suggest a benefit of adding immunotherapy to neoadjuvant chemotherapy with no significant increased surgical delay or risk.

**Table 4:** Benefits and risks of neoadjuvant immunotherapy.

Benefits	Risks
Treatment of micro-metastatic disease	Delayed surgery date with possibility of becoming unresectable
Better drug delivery in untreated tissue and vasculature	Potentially more difficult surgery due to immunotherapy effects on tissue
Increased patient adherence to treatment	Increased risk of post-operative morbidity
Ability to follow treatment response with pCR	More narrow patient selection
Ability to study resected tumor for markers of response	
Improved immunotherapy efficacy with higher antigen load	

pCR, pathologic complete response.

**Table 5** displays the patient characteristics that have been shown to confer an increased or decreased benefit of immunotherapy in these studies. Adding neoadjuvant immunotherapy to chemotherapy was shown in multiple trials (IMpower010, CheckMate816, KEYNOTE 671, NADIM II, etc.) to be especially beneficial in patients with PD-L1 expression ≥ 1%, due to positive correlation with survival. The benefit of histologic subtype was inconclusive in these studies, as both squamous and non-squamous histology were associated with increased responses from immunotherapy depending on the trial (81). A few studies showed no benefit of perioperative immunotherapy in patients who harbored an *EGFR* driver mutation. Neoadjuvant chemoimmunotherapy might also be less beneficial in patients with *STK11* tumor mutations, likely due to the *STK11* mutation being associated with reduced expression of the PD-L1 protein (81). Additionally, some trials also suggested immunotherapy may have limited benefit in patients without a smoking history. Many of these studies were insufficiently powered to make definitive conclusions from their subgroup analyses and further research is needed to better understand these associations.

**Table 5:** Characteristics affecting immunotherapy outcomes.

Greater IO Benefit	Less IO Benefit
Higher PD-L1 expression *(NEOSTAR, NADIM, CheckMate 816, AEGEAN, NADIM II, IMPOWER 010, KEYNOTE 091, CheckMate 77T)*	Targetable driver mutation *(NADIM, Columbia)*
More advanced stage *(CheckMate 816, AEGEAN, KEYNOTE 671, CheckMate 77T)*	*STK11* mutation *(Columbia, NADIM)*
Squamous histology *(ChiCTR-OIC-17013726, Columbia, CheckMate 77T)*	Non-smokers *(AEGEAN, KEYNOTE 671, CheckMate 77T)*
Non-squamous histology *(TOP1501, CheckMate 816)*	

IO, immunotherapy.

In patients with *EGFR* mutations, neoadjuvant TKIs alone had a minimal MPR rate (9.7% to 15%), with the EMERGING-CTONG 1103 and NCT03433469 trials showing no patients with PCR. However, they were able to downstage patients consistently in various trials (ranging from 35.7% to 44%). When given with radiotherapy in the ASCENT trial, MPR was 70% with a 2-year OS of 88%. This suggests that neoadjuvant TKIs may have additional benefit when used in combination with other neoadjuvant treatments and may be considered as neoadjuvant treatment in patients with targetable mutations with potentially resectable NSCLC. However, further studies are needed to evaluate neoadjuvant TKI usage, their ability to downstage potentially resectable patients, and whether the other common targetable driver mutations in NSCLC might confer the same results.

In the adjuvant setting, cisplatin doublet chemotherapy is the standard of care for stage IIIA NSCLC patients after surgical resection based on data from the LACE meta-analysis, which showed an 11% reduction in risk of death for all patients (HR 0.89; 95% CI, 0.82 to 0.96), with a 17% reduction in risk of death specifically in stage III patients (HR 0.83; 95% CI, 0.72 to 0.94) (82). In the adjuvant studies reviewed in this paper in which immunotherapy followed chemotherapy, the IMpower010 trial had a HR 0.66, while the KEYNOTE 091 trial showed an 18-month DFS benefit of 73.8% (compared to 63.1% with chemotherapy). Both these trials suggest an added benefit of adjuvant immunotherapy, but they were no subgroup analyses looking at stage IIIA disease and further research is needed regarding the timing of adjuvant immunotherapy. Whether to give sequential vs. concurrent treatment is currently being studied in the ACCIO trial. Concurrent adjuvant chemoimmunotherapy could be promising since some data has shown that chemotherapy mutation of tumor cells may make them more sensitive to concurrent immunotherapy and may improve outcomes (83).

Despite the encouraging data from the multiple trials reviewed in this paper, data is still immature regarding the ideal treatment plan for all newly diagnosed patients with stage IIIA-N2 NSCLC. This presents the main research gap in this field, which is related to the optimal sequencing of these multimodal therapies. This includes whether a patient with resectable, stage IIIA-N2 NSCLC should receive both neoadjuvant and adjuvant chemotherapy, immunotherapy, or TKI, and in which combination and which order.

In patients without driver mutations, neoadjuvant chemoimmunotherapy has been consistently shown to have superior outcomes compared with chemotherapy alone, even when focusing on stage III disease, so the former is now considered standard of care. Many studies have shown the benefit of neoadjuvant chemoimmunotherapy followed by adjuvant immunotherapy with good efficacy (NADIM, AEGEAN, CheckMate 77T). However, the IMpower010 and KEYNOTE 091 trials also show that adjuvant chemoimmunotherapy alone has improved outcomes which brings into question whether neoadjuvant therapy may even be needed. A head-to-head comparison is unlikely to be studied in NSCLC, but SWOG1801 did show that in melanoma, neoadjuvant and adjuvant immunotherapy had superior 2-year EFS compared to adjuvant immunotherapy alone (84). Another unanswered question is regarding the optimal duration of adjuvant immunotherapy since it is unclear whether there is an additional benefit of continuing immunotherapy beyond the traditional 1-2 years. Additionally, whether adding immunotherapy concurrently with adjuvant chemotherapy will further improve DFS is currently under investigation. Further subgroup analyses focusing on specific benefits in stage IIIA disease is also warranted.

In patients with driver mutations, we have more data supporting suboptimal rather than optimal treatment sequencing. Evidence shows that the risk of IRAEs increases when giving a TKI as subsequent therapy to an immunotherapeutic agent (85). We also have data suggesting that patients with driver mutations may be less likely to even benefit from perioperative immunotherapy (86). While some trials provide data suggesting neoadjuvant EGFR inhibitors may downstage potentially resectable patients, this is preliminary data and should be evaluated in future research. The current standard of care for these patients is to use neoadjuvant chemotherapy alone. In the adjuvant setting, further chemotherapy may be used, however recent studies have shown that patients with either an *EGFR* or *ALK* alteration may have improved DFS rates with adjuvant TKI therapy alone.

The sequencing of multimodal treatments also brings into question the increased toxicity risk of both neoadjuvant and adjuvant treatment. So far, most trials have acceptable levels of toxicity with minimal treatment-related deaths. Overall, research has shown a dramatic benefit with little excess risk when combining immunotherapy or TKIs in both the neoadjuvant and adjuvant settings of patients with stage IIIA-N2 NSCLC. Future research should explore the optimal sequence that maximizes efficacy and minimizes toxicity of these different options, especially in stage IIIA-N2 disease.

## Author contributions

MH: Writing – original draft, Writing – review & editing. SR: Supervision, Validation, Visualization, Writing – original draft, Writing – review & editing.

## References

[1] Group USCSW. U.S. Cancer Statistics Data Visualizations Tool, based on 2022 submission data (1999-2020) (2023). Available at: https://gis.cdc.gov/Cancer/USCS/#/AtAGlance/.

[2] SEER NIoH. Cancer Stat Facts: Lung and Bronchus Cancer. Available at: https://seer.cancer.gov/statfacts/html/lungb.html.

[3] Non-Small Cell Lung Cancer Stages: American Cancer Society (2019). Available at: https://www.cancer.org/cancer/types/lung-cancer/detection-diagnosis-staging/staging-nsclc.html.

[4] Non-Small Cell Lung Cancer (Version 5.2023): National Comprehensive Cancer Network (2023). Available at: https://www.nccn.org/professionals/physician_gls/pdf/nscl.pdf.

[5] Pataer A Weissferdt A Correa AM Vaporciyan AA Sepesi B Heymach JV. Major pathologic response and prognostic score predict survival in patients with lung cancer receiving neoadjuvant chemotherapy. JTO Clin Res Rep. (2022) 3:100420. doi: 10.1016/j.jtocrr.2022.100420 36389133 PMC9646962

[6] Mielgo-Rubio X Montemuiño S Jiménez U Luna J Cardeña A Mezquita L. Management of resectable stage III-N2 non-small-cell lung cancer (NSCLC) in the age of immunotherapy. Cancers. (2021) 13:4811. doi: 10.3390/cancers13194811 34638296 PMC8507745

[7] Velcheti V Schalper K. Basic overview of current immunotherapy approaches in cancer. Am Soc Clin Oncol Educ Book. (2016) 2016:298–308. doi: 10.1200/EDBK_156572 27249709

[8] Chae YK Arya A Iams W Cruz MR Chandra S Choi J. Current landscape and future of dual anti-CTLA4 and PD-1/PD-L1 blockade immunotherapy in cancer; lessons learned from clinical trials with melanoma and non-small cell lung cancer (NSCLC). J ImmunoTher Cancer. (2018) 6. doi: 10.1186/s40425-018-0349-3 PMC5956851 29769148

[9] Thomas A Rajan A Giaccone G. Tyrosine kinase inhibitors in lung cancer. Hematology/Oncol Clinics North America. (2012) 26:589–605. doi: 10.1016/j.hoc.2012.02.001 PMC3334853 22520981

[10] Sequist LV Neal JW. Personalized, genotype-directed therapy for advanced non-small cell lung cancer (2023). Available at: https://www.uptodate.com/contents/personalized-genotype-directed-therapy-for-advanced-non-small-cell-lung-cancer.

[11] Forde PM Chaft JE Smith KN Anagnostou V Cottrell TR Hellmann MD. Neoadjuvant PD-1 blockade in resectable lung cancer. New Engl J Med. (2018) 378:1976–86. doi: 10.1056/NEJMoa1716078 PMC6223617 29658848

[12] Bai R Li L Chen X Chen N Song W Cui J. Neoadjuvant and adjuvant immunotherapy: opening new horizons for patients with early-stage non-small cell lung cancer. Front Oncol. (2020) 10. doi: 10.3389/fonc.2020.575472 PMC7581706 33163406

[13] Gao S Li N Gao S Xue Q Ying J Wang S. Neoadjuvant PD-1 inhibitor (Sintilimab) in NSCLC. J Thorac Oncol. (2020) 15:816–26. doi: 10.1016/j.jtho.2020.01.017 32036071

[14] ODAC. FDA Briefing Document BLA 761222 Sintilimab. Silver Spring, MD: U.S. Food and Drug Administration (2022).

[15] Lee J Chaft J Nicholas A Patterson A Waqar S Toloza E. PS01.05 surgical and clinical outcomes with neoadjuvant atezolizumab in resectable stage IB–IIIB NSCLC: LCMC3 trial primary analysis. J Thorac Oncol. (2021) 16:S59–61. doi: 10.1016/j.jtho.2021.01.320

[16] Chaft JE Oezkan F Kris MG Bunn PA Wistuba II Kwiatkowski DJ. Neoadjuvant atezolizumab for resectable non-small cell lung cancer: an open-label, single-arm phase II trial. Nat Med. (2022) 28:2155–61. doi: 10.1038/s41591-022-01962-5 PMC9556329 36097216

[17] Wislez M Mazieres J Lavole A Zalcman G Carre O Egenod T. Neoadjuvant durvalumab for resectable non-small-cell lung cancer (NSCLC): results from a multicenter study (IFCT-1601 IONESCO). J ImmunoTher Cancer. (2022) 10:e005636. doi: 10.1136/jitc-2022-005636 36270733 PMC9594538

[18] Tong BC Gu L Wang X Wigle DA Phillips JD Harpole DH Jr.. Perioperative outcomes of pulmonary resection after neoadjuvant pembrolizumab in patients with non-small cell lung cancer. J Thorac Cardiovasc Surgery. (2022) 163:427–36. doi: 10.1016/j.jtcvs.2021.02.099 33985811

[19] National Library of Medicine. Neoadjuvant Anti PD-1 Immunotherapy in Resectable Non-small Cell Lung Cancer (NEOMUN). Bethesda, MD: ClinicalTrials.gov (2023). Available at: https://clinicaltrials.gov/study/NCT03197467.

[20] Cascone T William WN Weissferdt A Leung CH Lin HY Pataer A. Neoadjuvant nivolumab or nivolumab plus ipilimumab in operable non-small cell lung cancer: the phase 2 randomized NEOSTAR trial. Nat Med. (2021) 27:504–14. doi: 10.1038/s41591-020-01224-2 PMC8818318 33603241

[21] Watanabe S-I Nakagawa K Suzuki K Takamochi K Ito H Okami J. Neoadjuvant and adjuvant therapy for Stage III non-small cell lung cancer. Japanese J Clin Oncol. (2017) 47:1112–8. doi: 10.1093/jjco/hyx147 29136212

[22] Provencio M Nadal E Insa A García-Campelo MR Casal-Rubio J Dómine M. Neoadjuvant chemotherapy and nivolumab in resectable non-small-cell lung cancer (NADIM): an open-label, multicenter, single-arm, phase 2 trial. Lancet Oncol. (2020) 21:1413–22. doi: 10.1016/S1470-2045(20)30453-8 32979984

[23] Provencio M Serna-Blasco R Nadal E Insa A García-Campelo MR Casal Rubio J. Overall Survival and Biomarker Analysis of Neoadjuvant Nivolumab Plus Chemotherapy in Operable Stage IIIA Non–Small-Cell Lung Cancer (NADIM phase II trial). J Clin Oncol. (2022) 40:2924–33. doi: 10.1200/JCO.21.02660 PMC9426809 35576508

[24] Shu CA Gainor JF Awad MM Chiuzan C Grigg CM Pabani A. Neoadjuvant atezolizumab and chemotherapy in patients with resectable non-small-cell lung cancer: an open-label, multicenter, single-arm, phase 2 trial. Lancet Oncol. (2020) 21:P786–95. doi: 10.1016/S1470-2045(20)30140-6 32386568

[25] Rothschild SI Zippelius A Eboulet EI Savic Prince S Betticher D Bettini A. SAKK 16/14: durvalumab in addition to neoadjuvant chemotherapy in patients with stage IIIA(N2) non–small-cell lung cancer—A multicenter single-arm phase II trial. J Clin Oncol. (2021) 39:2872–80. doi: 10.1200/JCO.21.00276 34251873

[26] Forde PM Spicer J Lu S Provencio M Mitsudomi T Awad MM. Neoadjuvant nivolumab plus chemotherapy in resectable lung cancer. New Engl J Med. (2022) 386:1973–85. doi: 10.1056/NEJMoa2202170 PMC9844511 35403841

[27] Girard N. (2023). Neoadjuvant nivolumab (N) + platinum-doublet chemotherapy (C) for resectable NSCLC: 3-y update from CheckMate 816. Journal of Thoracic Oncology In: European Lung Cancer Congress, (Lugano, Switzerland: European Lung Cancer Congress).

[28] Bristol Myers Squibb. Neoadjuvant Opdivo (nivolumab) with chemotherapy provides benefits for patients with resectable non-small cell lung cancer across PD-L1 expression levels with three-year follow up in CheckMate-816 trial. Princeton, NJ: Business Wire (2023).

[29] U.S. Food and Drug Administration. FDA approves neoadjuvant nivolumab and platinum-doublet chemotherapy for early-stage non-small cell lung cancer. Silver Spring, MD: U.S. Food & Drug Administration (2022). Available at: https://www.fda.gov/drugs/resources-information-approved-drugs/fda-approves-neoadjuvant-nivolumab-and-platinum-doublet-chemotherapy-early-stage-non-small-cell-lung.

[30] Heymach JV Harpole D Mitsudomi T Taube JM Galffy G Hochmair M. Perioperative durvalumab for resectable non–small-cell lung cancer. New Engl J Med. (2023) 389:1672–84. doi: 10.1056/NEJMoa2304875 37870974

[31] Flaherty C. AEGEAN subgroup analysis fails to demonstrate clear benefit with perioperative durvalumab plus chemo in EGFR+ NSCLC. Cranbury, NJ: OncLive (2023). Available at: https://www.onclive.com/view/aegean-subgroup-analysis-fails-to-demonstrate-clear-benefit-with-perioperative-durvalumab-plus-chemo-in-egfr-nsclc?utm_source=www.onclive.com&utm_medium=relatedContent.

[32] Provencio M Nadal E González-Larriba JL Martínez-Martí A Bernabé R Bosch-Barrera J. Perioperative nivolumab and chemotherapy in stage III non–small-cell lung cancer. New Engl J Med. (2023) 389:504–13. doi: 10.1056/NEJMoa2215530 37379158

[33] Wakelee H Liberman M Kato T Tsuboi M Lee S-H Gao S. Perioperative pembrolizumab for early-stage non–small-cell lung cancer. New Engl J Med. (2023) 389:491–503. doi: 10.1056/NEJMoa2302983 37272513 PMC11074923

[34] Kahl KL. Perioperative nivolumab significantly improves EFS in previously untreated resectable NSCLC. Cranbury, NJ: OncLive (2023). Available at: https://www.onclive.com/view/perioperative-nivolumab-significantly-improves-efs-in-previously-untreated-resectable-nsclc.

[35] Goodman A. Perioperative nivolumab plus chemotherapy improves event-free survival in resectable non-small cell lung cancer. Huntington, NY: The ASCO Post (2023). Available at: https://ascopost.com/issues/december-10-2023-supplement-esmo-highlights/perioperative-nivolumab-plus-chemotherapy-improves-event-free-survival-in-resectable-non-small-cell-lung-cancer/.

[36] Cascone T Awad MM Spicer JD He J Lu S Sepesi B. LBA1 CheckMate 77T: Phase III study comparing neoadjuvant nivolumab (NIVO) plus chemotherapy (chemo) vs neoadjuvant placebo plus chemo followed by surgery and adjuvant NIVO or placebo for previously untreated, resectable stage II–IIIb NSCLC. Ann Oncol. (2023) 34:S1295. doi: 10.1016/j.annonc.2023.10.050

[37] National Library of Medicine. A study of neoadjuvant atezolizumab plus chemotherapy versus placebo plus chemotherapy in patients with resectable stage II, IIIA, or select IIIB non-small cell lung cancer (IMpower030). Bethesda, MD: ClinicalTrials.gov (2023). Available at: https://clinicaltrials.gov/study/NCT03456063.

[38] Altorki NK McGraw TE Borczuk AC Saxena A Port JL Stiles BM. Neoadjuvant durvalumab with or without stereotactic body radiotherapy in patients with early-stage non-small-cell lung cancer: a single-center, randomized phase 2 trial. Lancet Oncol. (2021) 22:P824–35. doi: 10.1016/S1470-2045(21)00149-2 34015311

[39] National Library of Medicine. Neoadjuvant chemoradiation plus pembrolizumab followed by consolidation pembrolizumab in NSCLC. Bethesda, MD: ClinicalTrials.gov (2022). Available at: https://clinicaltrials.gov/study/NCT02987998.

[40] National Library of Medicine. Neoadjuvant immunoradiation for resectable non-small cell lung cancer. Bethesda, MD: ClinicalTrials.gov (2023). Available at: https://clinicaltrials.gov/study/NCT03237377.

[41] National Library of Medicine. Multimodality treatment in stage III non-small cell lung cancer (NSCLC). Bethesda, MD: ClinicalTrials.gov (2023). Available at: https://clinicaltrials.gov/study/NCT04245514.

[42] Xiong L Li R Sun J Lou Y Zhang W Bai H. Erlotinib as neoadjuvant therapy in stage IIIA (N2) EGFR mutation-positive non-small cell lung cancer: A prospective, single-arm, phase II study. Oncologist. (2019) 24:157–e64. doi: 10.1634/theoncologist.2018-0120 30158288 PMC6369937

[43] Zhong W-Z Chen K-N Chen C Gu C-D Wang J Yang X-N. Erlotinib versus gemcitabine plus cisplatin as neoadjuvant treatment of stage IIIA-N2 EGFR-mutant non–small-cell lung cancer (EMERGING-CTONG 1103): A randomized phase II study. J Clin Oncol. (2019) 37:2235–45. doi: 10.1200/JCO.19.00075 31194613

[44] Leighl NB Karaseva N Nakagawa K Cho B-C Gray JE Hovey T. Patient-reported outcomes from FLAURA: Osimertinib versus erlotinib or gefitinib in patients with EGFR-mutated advanced non-small-cell lung cancer. Eur J Cancer. (2020) 125:49–57. doi: 10.1016/j.ejca.2019.11.006 31838405

[45] Piper-Vallillo A Mak R Lanuti M Muzikansky A Rotow J Jänne P. FP01.05 the ASCENT trial: A phase II study of neoadjuvant/adjuvant afatinib, chemoradiation +/- surgery for stage III EGFR-mutant NSCLC. J Thorac Oncol. (2021) 16:S188. doi: 10.1016/j.jtho.2021.01.072 PMC11224994 38761385

[46] Targeted Oncology. Afatinib shows encouraging efficacy and safety in late stage EGFR-mutant NSCLC. Cranbury, NJ: Targeted Oncology (2021). Available at: https://www.targetedonc.com/view/afatinib-shows-encouraging-efficacy-and-safety-in-late-stage-egfr-mutant-nsclc.

[47] Lv C Fang W Wu N Jiao W Xu S Ma H. Osimertinib as neoadjuvant therapy in patients with EGFR-mutant resectable stage II-IIIB lung adenocarcinoma (NEOS): A multicenter, single-arm, open-label phase 2b trial. Lung Cancer. (2023) 178:151–6. doi: 10.1016/j.lungcan.2023.02.011 36863124

[48] Aredo JV Urisman A Gubens MA Mulvey C Allen GM Rotow JK. Phase II trial of neoadjuvant osimertinib for surgically resectable EGFR-mutated non-small cell lung cancer. J Clin Oncol. (2023) 41:8508. doi: 10.1200/JCO.2023.41.16_suppl.8508

[49] Aredo JV Urisman A Gubens MA Mulvey C Allen GM Rotow JK. (2023). Phase II trial of neoadjuvant osimertinib for surgically resectable EGFR-mutated non-small cell lung cancer. Journal of Clinical Oncology in: 2023 ASCO Annual Meeting, (Alexandria, VA: American Society of Clinical Oncology). doi: 10.1200/JCO.2023.41.16_suppl.8508

[50] Lovely B. Neoadjuvant osimertinib yield promising benefit in EGFR-positive non-small cell lung cancer. Cancer Network. (2021) (Cranbury, NJ: MJH Life Sciences).

[51] Tsuboi M Weder W Escriu C Blakely C He J Dacic S. Neoadjuvant osimertinib with/without chemotherapy versus chemotherapy alone for EGFR-mutated resectable non-small-cell lung cancer: NeoADAURA. Future Oncol. (2021) 17:4045–55. doi: 10.2217/fon-2021-0549 PMC8530153 34278827

[52] National Library of Medicine. A study of osimertinib with or without chemotherapy versus chemotherapy alone as neoadjuvant therapy for patients with EGFRm positive resectable non-small cell lung cancer (NeoADAURA). Bethesda, MD: ClinicalTrials.gov (2023). Available at: https://clinicaltrials.gov/study/NCT04351555?tab=table.

[53] National Library of Medicine. A study of multiple therapies in biomarker-selected patients with resectable stages IB-III non-small cell lung cancer. Bethesda, MD: National Library of Medicine. Available at: https://clinicaltrials.gov/study/NCT04302025.

[54] Felip E Altorki N Zhou C Csoszi T Vynnychenko I Goloborodko O. Adjuvant atezolizumab after adjuvant chemotherapy in resected stage IB-IIIA non-small-cell lung cancer (IMpower010): a randomized, multicenter, open-label, phase 3 trial. Lancet. (2021) 398:1344–57. doi: 10.1016/S0140-6736(21)02098-5 34555333

[55] Felip E Altorki N Zhou C Vallières E Martínez-Martí A Rittmeyer A. Overall survival with adjuvant atezolizumab after chemotherapy in resected stage II-IIIA non-small-cell lung cancer (IMpower010): a randomized, multicenter, open-label, phase III trial. Ann Oncol. (2023) 34:907–19. doi: 10.1016/j.annonc.2023.07.001 37467930

[56] U.S. Food and Drug Administration. FDA approves atezolizumab as adjuvant treatment for non-small cell lung cancer. Silver Spring, MD: U.S. Food & Drug Administration (2023). Available at: https://www.fda.gov/drugs/resources-information-approved-drugs/fda-approves-atezolizumab-adjuvant-treatment-non-small-cell-lung-cancer.

[57] Oselin K Shim BY Okada M Bryl M Bonanno L Demirag G. Pembrolizumab vs placebo for early-stage non−small-cell lung cancer after resection and adjuvant therapy: Subgroup analysis of patients who received adjuvant chemotherapy in the phase 3 PEARLS/KEYNOTE-091 study. J Clin Oncol. (2023) 41:8520. doi: 10.1200/JCO.2023.41.16_suppl.8520

[58] O’Brien M Paz-Ares L Marreaud S Dafni U Oselin K Havel L. Pembrolizumab versus placebo as adjuvant therapy for completely resected stage IB-IIIA non-small-cell lung cancer (PEARLS/KEYNOTE-091): an interim analysis of a randomized, triple-blind, phase 3 trial. Lancet Oncol. (2022) 23:1274–86. doi: 10.1016/S1470-2045(22)00518-6 36108662

[59] U.S. Food and Drug Administration. FDA approves pembrolizumab as adjuvant treatment for non-small cell lung cancer. Silver Spring, MD: U.S. Food & Drug Administration (2023). Available at: https://www.fda.gov/drugs/resources-information-approved-drugs/fda-approves-pembrolizumab-adjuvant-treatment-non-small-cell-lung-cancer.

[60] National Library of Medicine. Phase III study to determine the efficacy of durvalumab in combination with chemotherapy in completely resected stage II-III non-small cell lung cancer (NSCLC) (MERMAID-1). Bethesda, MD: ClinicalTrials.gov (2023). Available at: https://clinicaltrials.gov/study/NCT04385368.

[61] National Library of Medicine. Testing the addition of a type of drug called immunotherapy to the usual chemotherapy treatment for non-small cell lung cancer, ALCHEMIST trial. Bethesda, MD: ClinicalTrials.gov (2023). Available at: https://clinicaltrials.gov/study/NCT04267848.

[62] National Library of Medicine. Nivolumab after surgery and chemotherapy in treating patients with stage IB-IIIA non-small cell lung cancer (An ALCHEMIST treatment trial) (ANVIL). Bethesda, MD: ClinicalTrials.gov (2023). Available at: https://clinicaltrials.gov/study/NCT02595944.

[63] National Library of Medicine. Double blind placebo controlled study of adjuvant MEDI4736 in completely resected NSCLC. Bethesda, MD: ClinicalTrials.gov (2023). Available at: https://clinicaltrials.gov/study/NCT02273375.

[64] Herbst RS Wu Y-L John T Grohe C Majem M Wang J. Adjuvant osimertinib for resected EGFR-mutated stage IB-IIIA non–small-cell lung cancer: updated results from the phase III randomized ADAURA trial. J Clin Oncol. (2023) 41:1830–40. doi: 10.1200/JCO.22.02186 PMC10082285 36720083

[65] Herbst RS Tsuboi M John T Kato T Majem M Grohé C. Overall survival analysis from the ADAURA trial of adjuvant osimertinib in patients with resected EGFR-mutated (EGFRm) stage IB–IIIA non-small cell lung cancer (NSCLC). J Clin Oncol. (2023) 41:LBA3–LBA. doi: 10.1200/JCO.2023.41.17_suppl.LBA3 PMC10082285 36720083

[66] American Society of Clinical Oncology. Final ADAURA OS analysis reinforces adjuvant osimertinib as a standard of care for patients with stage IB to IIIA EGFR-mutated non-small cell lung cancer. Alexandria, VA: ASCO Daily News (2023). Available at: https://dailynews.ascopubs.org/do/final-adaura-os-analysis-reinforces-adjuvant-osimertinib-standard-care-patients-stage.

[67] U.S. Food and Drug Administration. FDA approves osimertinib as adjuvant therapy for non-small cell lung cancer with EGFR mutations. Silver Spring, MD: U.S. Food & Drug Administration (2020). Available at: https://www.fda.gov/drugs/resources-information-approved-drugs/fda-approves-osimertinib-adjuvant-therapy-non-small-cell-lung-cancer-egfr-mutations.

[68] National Library of Medicine. A study of 5 years of adjuvant osimertinib in completely resected epidermal growth factor receptor mutation (EGFRm) non-small cell lung carcinoma (NSCLC) (TARGET). Silver Spring, MD: ClinicalTrials.gov (2023). Available at: https://www.clinicaltrials.gov/study/NCT05526755.

[69] National Library of Medicine. A study comparing adjuvant alectinib versus adjuvant platinum-based chemotherapy in patients with ALK positive non-small cell lung cancer. Bethesda, MD: ClinicalTrials.gov (2023). Available at: https://clinicaltrials.gov/study/NCT03456076.

[70] European Society for Medical Oncology. Adjuvant alectinib is a new treatment strategy for resected ALK-positive NSCLC. Lugano, Switzerland: ESMO Daily Reporter (2023). Available at: https://dailyreporter.esmo.org/esmo-congress-2023/non-small-cell-lung-cancer/adjuvant-alectinib-is-a-new-treatment-strategy-for-resected-alk-positive-nsclc.

[71] National Cancer Institute. The ALCHEMIST lung cancer trials. Bethesda, MD: National Cancer Institute (2017). Available at: https://www.cancer.gov/types/lung/research/alchemist.

[72] National Library of Medicine. Crizotinib in treating patients with stage IB-IIIA non-small cell lung cancer that has been removed by surgery and ALK fusion mutations (An ALCHEMIST treatment trial). Bethesda, MD: ClinicalTrials.gov (2023). Available at: https://clinicaltrials.gov/study/NCT02201992.

[73] Lung Cancer Research Foundation. FDA approvals in lung cancer treatment. New York, NY: Lung Cancer Research Foundation (2023). Available at: https://www.lungcancerresearchfoundation.org/research/why-research/treatment-advances/.

[74] Hellmann MD Chaft JE William WN Rusch V Pisters KMW Kalhor N. Pathological response after neoadjuvant chemotherapy in resectable non-small-cell lung cancers: proposal for the use of major pathological response as a surrogate endpoint. Lancet Oncol. (2014) 15:e42–50. doi: 10.1016/S1470-2045(13)70334-6 PMC4734624 24384493

[75] Fiteni F Westeel V Bonnetain F. Surrogate endpoints for overall survival in lung cancer trials: a review. Expert Rev Anticancer Ther. (2017) 17:447–54. doi: 10.1080/14737140.2017.1316196 28399678

[76] Cao C Le A Bott M Yang C-FJ Gossot D Melfi F. Meta-analysis of neoadjuvant immunotherapy for patients with resectable non-small cell lung cancer. Curr Oncol. (2021) 28:4686–701. doi: 10.3390/curroncol28060395 PMC8628782 34898553

[77] Roth JA Fossella F Komaki R Ryan MB Putnam JB Jr. Lee JS. A randomized trial comparing perioperative chemotherapy and surgery with surgery alone in resectable stage IIIA non-small-cell lung cancer. JNCI: J Natl Cancer Institute. (1994) 86:673–80. doi: 10.1093/jnci/86.9.673 8158698

[78] Liu J Blake SJ Yong MCR Harjunpää H Ngiow SF Takeda K. Improved efficacy of neoadjuvant compared to adjuvant immunotherapy to eradicate metastatic disease. Cancer Discovery. (2016) 6:1382–99. doi: 10.1158/2159-8290.CD-16-0577 27663893

[79] Bilusic M. What are the advantages of neoadjuvant immunotherapy over adjuvant immunotherapy? Expert Rev Anticancer Ther. (2022) 22:561–3. doi: 10.1080/14737140.2022.2069097 35473572

[80] Kang J Zhang C Zhong WZ. Neoadjuvant immunotherapy for non–small cell lung cancer: State of the art. Cancer Commun. (2021) 41:287–302. doi: 10.1002/cac2.12153 PMC8045926 33689225

[81] Dziadziuszko R. STK11 and KEAP1 mutations in lung adenocarcinoma: solving the puzzle continues. J Thorac Oncol. (2022) 17:351–2. doi: 10.1016/j.jtho.2022.01.004 35216730

[82] Pignon J-P Tribodet H Scagliotti GV Douillard J-Y Shepherd FA Stephens RJ. Lung adjuvant cisplatin evaluation: A pooled analysis by the LACE collaborative group. J Clin Oncol. (2008) 26:3552–9. doi: 10.1200/JCO.2007.13.9030 18506026

[83] Guo H Li W Qian L Cui J. Clinical challenges in neoadjuvant immunotherapy for non-small cell lung cancer. Chin J Cancer Res. (2021) 33:203–15. doi: 10.21147/j.issn.1000-9604.2021.02.08 PMC8181868 34158740

[84] Patel SP Othus M Chen Y Wright GP Yost KJ Hyngstrom JR. Neoadjuvant–adjuvant or adjuvant-only pembrolizumab in advanced melanoma. New Engl J Med. (2023) 388:813–23. doi: 10.1056/NEJMoa2211437 PMC10410527 36856617

[85] Schoenfeld AJ Arbour KC Rizvi H Iqbal AN Gadgeel SM Girshman J. Severe immune-related adverse events are common with sequential PD-(L)1 blockade and osimertinib. Ann Oncol. (2019) 30:839–44. doi: 10.1093/annonc/mdz077 PMC7360149 30847464

[86] Mazieres J Drilon A Lusque A Mhanna L Cortot AB Mezquita L. Immune checkpoint inhibitors for patients with advanced lung cancer and oncogenic driver alterations: results from the IMMUNOTARGET registry. Ann Oncol. (2019) 30:1321–8. doi: 10.1093/annonc/mdz167 PMC7389252 31125062

